# CDBA: a novel multi-branch feature fusion model for EEG-based emotion recognition

**DOI:** 10.3389/fphys.2023.1200656

**Published:** 2023-07-20

**Authors:** Zhentao Huang, Yahong Ma, Jianyun Su, Hangyu Shi, Shanshan Jia, Baoxi Yuan, Weisu Li, Jingzhi Geng, Tingting Yang

**Affiliations:** ^1^ School of Electronic Information, Xijing University, Xi’an, China; ^2^ Department of Neurosurgery, Affiliate Children’s Hospital of Xi’an Jiaotong University, Xi’an, China; ^3^ Department of Neurology, Affiliate Children’s Hospital of Xi’an Jiaotong University, Xi’an, China; ^4^ Graduate Student Institute of Xi’an Medical University, Xi’an, Shanxi Province, China

**Keywords:** convolution neural network (CNN), depthwise separable convolution (DSC), electroencephalogram (EEG), bi-directional long short term memory (Bi-LSTM), attention mechanism, emotion recognition

## Abstract

EEG-based emotion recognition through artificial intelligence is one of the major areas of biomedical and machine learning, which plays a key role in understanding brain activity and developing decision-making systems. However, the traditional EEG-based emotion recognition is a single feature input mode, which cannot obtain multiple feature information, and cannot meet the requirements of intelligent and high real-time brain computer interface. And because the EEG signal is nonlinear, the traditional methods of time domain or frequency domain are not suitable. In this paper, a CNN-DSC-Bi-LSTM-Attention (CDBA) model based on EEG signals for automatic emotion recognition is presented, which contains three feature-extracted channels. The normalized EEG signals are used as an input, the feature of which is extracted by multi-branching and then concatenated, and each channel feature weight is assigned through the attention mechanism layer. Finally, Softmax was used to classify EEG signals. To evaluate the performance of the proposed CDBA model, experiments were performed on SEED and DREAMER datasets, separately. The validation experimental results show that the proposed CDBA model is effective in classifying EEG emotions. For triple-category (positive, neutral and negative) and four-category (happiness, sadness, fear and neutrality), the classification accuracies were respectively 99.44% and 99.99% on SEED datasets. For five classification (Valence 1—Valence 5) on DREAMER datasets, the accuracy is 84.49%. To further verify and evaluate the model accuracy and credibility, the multi-classification experiments based on ten-fold cross-validation were conducted, the elevation indexes of which are all higher than other models. The results show that the multi-branch feature fusion deep learning model based on attention mechanism has strong fitting and generalization ability and can solve nonlinear modeling problems, so it is an effective emotion recognition method. Therefore, it is helpful to the diagnosis and treatment of nervous system diseases, and it is expected to be applied to emotion-based brain computer interface systems.

## 1 Introduction

EEG is the overall reflection of nerve cells in the cerebral cortex or scalp surface of electrophysiological activities in the brain, which has the advantages of being non-invasive, easy to use, portable, and other advantages. It is often used in sleep staging, seizure detection and prediction, brain-computer interfaces (BCIs) and other fields. Data-driven machine learning or deep learning can simulate or even improve the clinical diagnosis of EEG. Emotion is an important part of human life, which reflects human emotions, thoughts, behaviors and further affects physical and behavioral states (Lu et al., 2020). Positive emotions can promote and enhance, while negative emotions can weaken and reduce human behavior. Severe negative emotion, such as depression, may cause serious damage to the physical and mental health of patients. Emotion plays a crucial role in all aspects, such as human communication and decision-making. Therefore, understanding and analyzing human emotions is very important, which can be used in many fields, such as intelligent driving systems (Nakisa et al., 2018), medical services (Mehmood et al., 2017), voice assistants (Liu et al., 2017), robots (Dai et al., 2017) etc., When treating patients with emotional problems, auto-recognition of real emotional states can help doctor to provide better medical care. Several psychological and physiological studies have found a strong correlation between emotion recognition and brain activity (Sammler et al., 2007; Mathersul et al., 2008; Knyazev et al., 2010). Emotion can be recognized through physical activity, body posture, speech, facial expression, etc. However, these external signs are easy to be disguised or concealed, great impact by subjective factors and it is difficult to reflect the true emotional state of the heart. Physiological signals have the advantages of universality, spontaneity and difficulty in disguising, which can reflect the real emotional state more accurately.

Physiological signals can reflect the state of the central and autonomic nervous systems (CNS and ANS) (Cannon, 1927) and indicate a subject’s potential emotional response (Shu et al., 2018). With advanced mobile computing technologies and miniaturized wearable sensors, physiological signals can be continuously monitored, which include electroencephalogram (EEG), electrocardiogram (ECG), electromyogram (EMG), electrodermal response (GSR), body temperature, respiratory rate (RR), and pulse blood oxygen measurement. EEG signals have been shown to provide important features for emotion recognition (Petrantonakis and Hadjileontiadis, 2011), which has excellent spatial and temporal resolution during emotional induction (Dale and Sereno, 1993). EEG has the advantage of non-invasive, fast, and economic. However, the complexity and nonlinearity of EEG signals lead to the suboptimal processing effect of many traditional methods, the steps of which contains feature extraction and classification. According to the EEG signal characteristics, obtaining the most prominent features is the most critical step. Common feature extraction methods include time domain, frequency domain, time-frequency analysis, and nonlinear analysis, etc. However, traditional machine learning methods require selecting electrode channels and manually extracting features, which are tedious, time-consuming and laborious, and easily lead to low accuracy of classification results. Deep learning has the advantages of strong learning ability and portability (Chen et al., 2021), thus EEG signal recognition and classification based on deep learning has become an important research direction. Jaiswal and Banka, (2017) proposed an artificial neural networks (ANN) model for feature-based EEG signal classification, which used local gradient patterns and neighborhood description patterns, and the final classification accuracy is 99.82%. Zhang et al. (2018) proposed a Spatial Temporal Recursive Neural Network (STRNN) for emotion recognition, the results of which shows that STRNN performed significantly better than SVM. Zheng and Lu (2015) used a short Fourier Transform to extract density entropy of multichannel EEG signals and used Deep Trust Network (DBN) to classify positive, negative, and neutral emotions with 86.65% accuracy.

Many researchers have devoted themselves to put forward various algorithms to improve the accuracy of emotion classification. For example, studies that selected representative spatial and temporal information can greatly optimize the classification of emotion, and studies that selected a minimum number of channels to determine the emotional state of EEG signals without compressing accuracy can be applied to portable EEG interface implants. EEG signals are time-varying and continuous, which is very useful for classifying emotional states. Therefore, a good combination of spatial temporal combination features can provide more information.

According to the characteristics of EEG signals and the shortcomings of existing methods, this paper proposes a CNN-DSC-Bi-LSTM-Attention (CDBA) model based on multi-branching feature fusion, which can achieve the feature extraction, feature selection and classification for emotional EEG. There are three channels to extract spatial and temporal features, including primary spatial features and advanced spatial features. According to the importance of EEG features, attention mechanisms are used to give different weights to each feature. Finally, Softmax function is used as a classifier to classify emotions and obtain triple or four categories results. Compared to existing research, the innovations of this paper can be summarized as follows:

(1) The use of raw EEG signals facilitates transplantation and application to brain interfaces.
(2) Bi-LSTM, Depthwise Separable Convolution (DSC), and Attention Mechanism are introduced to achieve high performance and lower model overhead by combining multiple features.
(3) Extensive experiments were conducted on both SEED datasets to verify and evaluate the model accuracy and credibility.

The organizational structure of this paper is as follows: Section 2 describes the details of the data set, the classification model architecture, and the overall framework of this paper. Section 3 introduces the results of this paper and compares them with other models. Section 4 summarizes this paper and puts forward the future research direction.

## 2 Materials and methods

### 2.1 Dataset

SJTU Emotion EEG Dataset (SEED) is provided by the BCMI Laboratory of Shanghai Jiaotong University (Duan et al., 2013; Zheng and Lu, 2015). The data of SEED was from EEG recordings of 15 subjects. During the experiment, 15 Chinese film clips (positive, neutral, and negative emotions) were selected from a library of materials as stimuli used in the experiment. The total duration of the experiment should be short. Otherwise, subjects may feel tired. Film clips should be well understood, not clarified. Clips should stimulate single-goal emotions. It shouldn't contain mixed emotions. Each film has been edited to create coherent emotion and maximize emotional meaning. There were a total of 15 tests per experiment. Each clip is preceded by a 5-s reminder, 45 s for self-assessment, and 15 s to rest after each clip in a session. The order of screenings is such that two clips of the same emotion will not be shown consecutively. In response to feedback, participants were told to report their emotional reaction to each film clip by completing a questionnaire immediately after viewing each clip. SEED-IV (Zheng et al., 2018) was data from EEG recordings of 15 subjects, and 72 film clips were carefully selected for three experiments, which tended to induce feelings of happiness, sadness, fear, or neutrality, as SEED did. Table 1 gives some details of the film clips used in the SEED experiment.

**TABLE 1:** Examples of the movie clips of Positive, Negative and Neutral emotions (Duan et al., 2013; Zheng and Lu, 2015; Zheng et al., 2018).

Serial No	Emotion label	Film clips’ sources
01	Negative	Back to 1942
02	Negative	Tangshan Earthquake
03	Positive	Flirting Scholar
04	Positive	Lost in Thailand
05	Neutral	World Heritage in China
06	Neutral	A Bite of China

Similarly, the DREAMER database (Gabert-Quillen et al., 2015; Katsigiannis and Ramzan, 2017) consists of multichannel EEG signals collected from 23 healthy subjects (9 women and 14 men). In this database, each subject’s mood was induced by playing 18 movie clips. Each film clip considered nine emotional categories, such as anger, fear, calm, entertainment, sadness, surprise, disgust, happiness, and excitement. EEG signals were recorded with 14 electrodes (using a standard 10–20 electrode system) and sampled at 128 Hz.

### 2.2 Data pre-processing

The SEED dataset was tested in three stages for each subject. The interval between each experiment was 1 week or more. This process ensures a stable pattern of neural activity at different stages and in different individuals. Record both facial video and EEG. Subjects sat in front of large screens showing movie clips. The data were collected via 62 channels which are placed according to 10–20 system, down-sampled to 200 Hz, a bandpass frequency filter from 0–75 Hz was applied and presented as MATLAB “.mat” files. In total, 45 sessions of EEG data was recorded. The labels are given according to the clip contents (−1 for negative, 0 for neutral and 1 for positive). To test the superiority of the model for emotional recognition, we used only the original EEG signals of SEED and SEED-IV for test training. Due to the large volume of data, we extracted 1,000 consecutive datasets from each person in the experiment in the middle of each video segment, so SEED and SEED-IV were extracted for 15 people × 15 videos × 1,000 = 225,000 and 15 people × 24 videos × 1,000 = 360,000.

The same DREAMER was created by 23 subjects each watching 18 movie clips, producing 23 × 18 = 414 EEG files. Each subject was given a score between 1 and 5 based on valence, arousal, and dominance levels, and the Valence classifications was used as a label in the paper. We extract 1,000 consecutive data sets from each person in the experiment in the middle of each video clip, so the extraction DREAMER is 23 people x 18 videos × 1,000 = 414,000.

### 2.3 The structure of CDBA model

**FIGURE 1:**
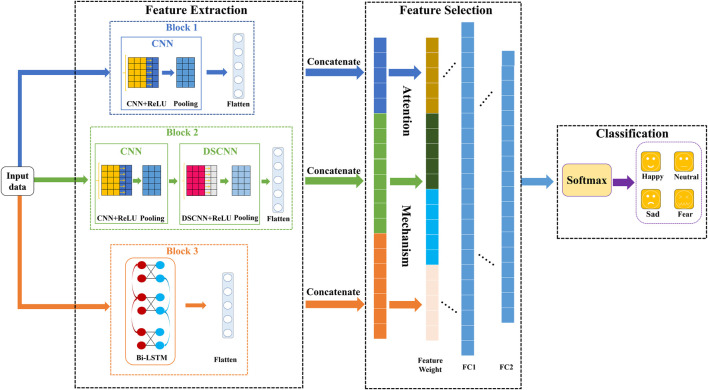
CDBA structure.

The importance of each feature in the emotion classification is different, a CDBA model for emotion recognition is proposed in this paper. The structure of the CDBA model is shown in Figure 1.

It can be seen from Figure 1, the CDBA model can achieve the feature extraction, feature selection and classification for emotional EEG. There are three parallel channels to extract spatial and temporal features, including primary spatial features and advanced spatial features. The raw EEG signals are first normalized and then input simultaneously to the three Blocks. Block 1 consists of a convolution layer with ReLU activation function, a max pooling layer, and a flatten layer. The reason for choosing ReLU activation function is that it abandons complex computation to improve the operation speed, which helps to solve the convergence problem of deep networks. And it is more in line with the characteristics of biological neurons and is easy to learn and optimize. Block 2 added a deeply separable network to Block 1. Depth separable convolution (DSCNN) can be divided into deep and separable convolution, mainly obtains the interaction information between physiological signal space and characteristic channels. In the depth convolution part, all the connected channels of each sample point are convolved. In the separable convolution part, each sample point channel convolution is performed on the basis of deep convolution. 64 unit Bi-LSTM and flatten layers are used in Block 3, which is solves the long term dependence of cyclic neural network RNN. The unique “gate” structure avoids gradient explosion and gradient disappearance and has the advantage of strong long term memory. Bi-LSTM considers forward and backward time series information in the time dimension to make the prediction more comprehensive and accurate. CNN is suitable for extracting local spatial features, and Bi-LSTM combines bidirectional time-series information to analyze emotional characteristics more thoroughly from spatial-temporal characteristics and improve the fit of predictive results. The primary spatial features, the advanced spatial features and the time series features are extracted from the three blocks, respectively. And then they are concatenated together into a sequence. The attention mechanism layer will weigh the concatenated sequence, assign weight values to each channel, further extract features and reduce dimensions through two fully connected layers, and then categorize the final prediction results through the Softmax function. Parameters of the CDBA architecture are shown in Table 2.

**TABLE 2:** Parameters of the CDBA architecture.

	Type	Number	Size
Block 1	Conv1d	32	3 size*1 Step size
	MaxPooling 1d		2 size*1 Step size
Block 2	Separable_Conv1d	32	3 size*1 Step size
	MaxPooling 1d		2 size*1 Step size
	Conv1d	32	3 size*1 Step size
	MaxPooling1d		2 size*1 Step size
Block 3	Bi-LSTM	64	
FC1	Dense	64	
FC2	Dense	32	

#### 2.3.1 Convolution neural network

**FIGURE 2:**
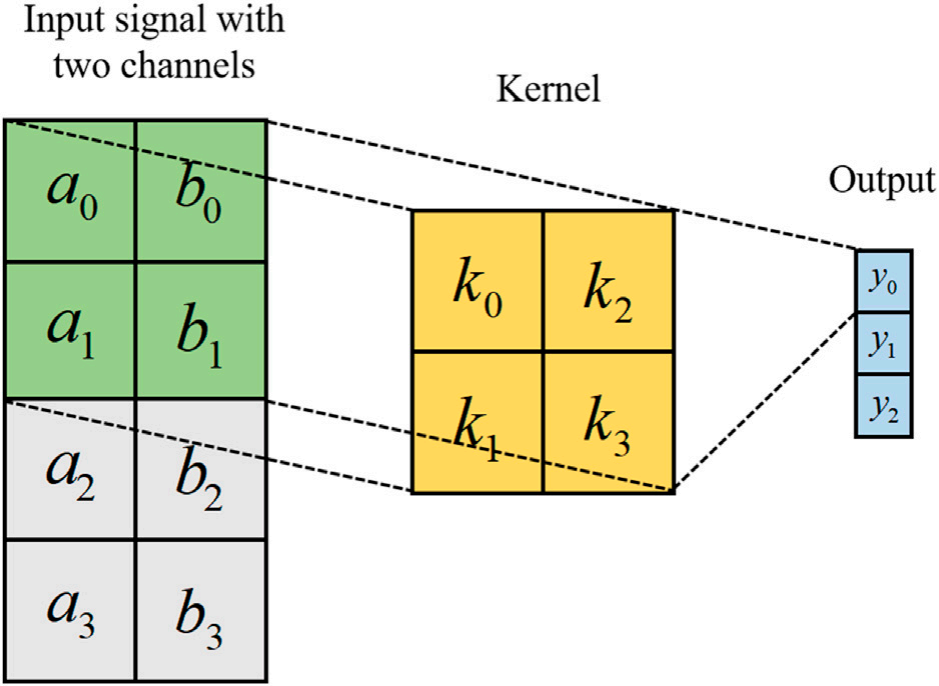
Two channels one-dimensional convolution.

CNN is a deep learning model that automatically learns to classify objects from images, numbers or videos, which generally consists of convolution, pooling and full connection layers. The convolution layer contains 32 convolution nuclei and performs convolution calculations on input signals. Then the non-linearization of the convolution results is performed by using the activation function. By optimizing the weight parameters of each filter, the algorithm minimizes classification errors and can learn from input data. The EEG data used in this paper is one-dimensional, so only one-dimensional convolution neural networks are used, the diagram of which is shown in Figure 2. Rectifying linear activation units (ReLU) were used in the one-dimensional convolution layer. The pooling layer, also known as the down-sampling layer, performs pool operations on the output of the convolution layer to preserve higher-level representation. The advanced features are usually fed into the full connection layer for final classification. Assuming the input is two-channel data, the kernel is also a two-dimensional two-column filter, with the first convolution output calculated as Eq. 1.

$$y_{0}=a_{0}\times k_{0}+a_{1}\times k_{1}+b_{0}\times k_{2}+b_{1}\times k_{3}$$ (1)

The rest of the output is calculated by sliding the kernel in a vertical direction, that is, time-stamp direction. Thus, vectors can be obtained from each filter, which is connected by columns to obtain a two-dimensional feature diagram and further processed by continuous convolution operations. Through multiple convolution operations until the abstract features of the signal are extracted.

#### 2.3.2 Depthwise separable convolution

**FIGURE 3:**
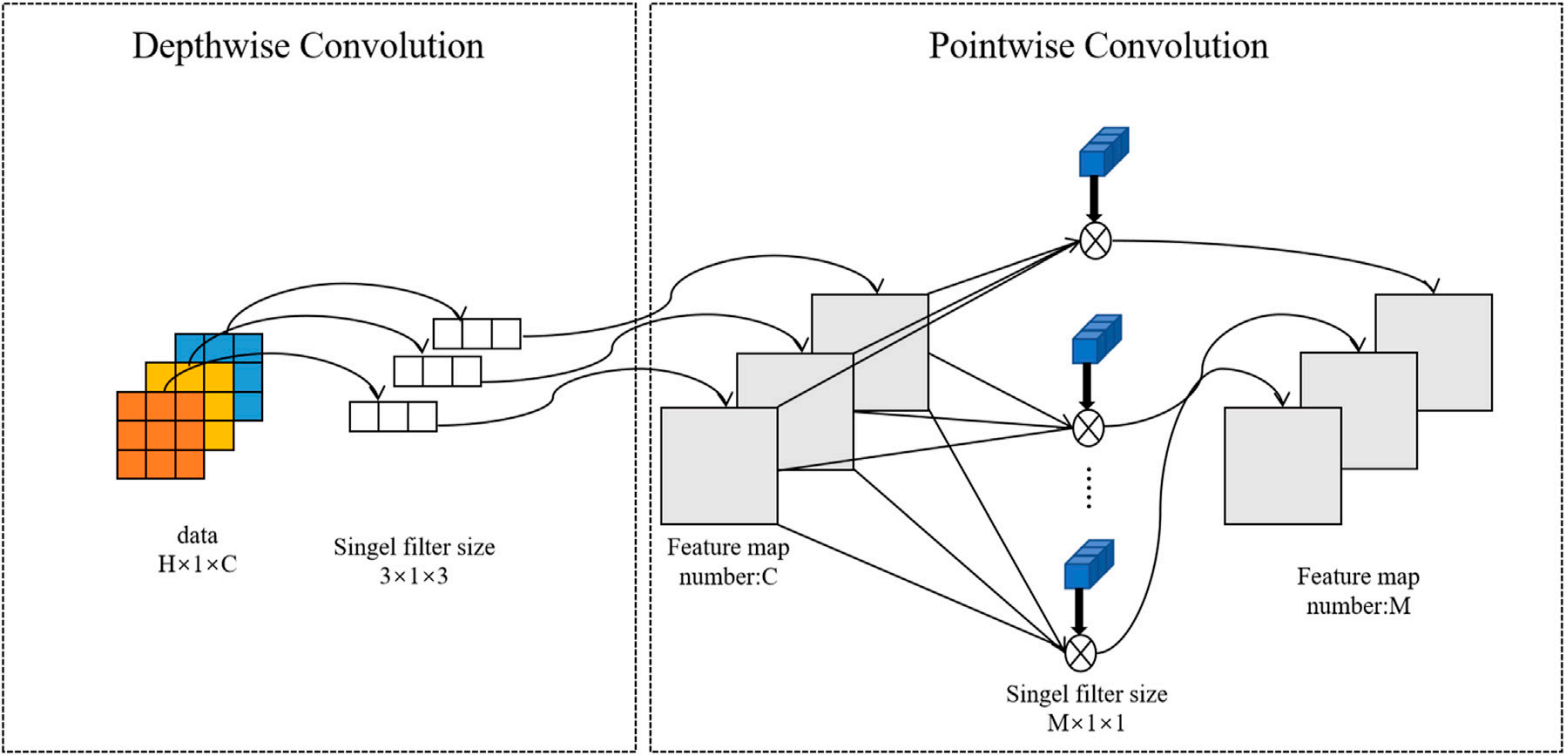
Convolution process of one-dimensional depthwise separable convolution.

DSCNN can be divided into depthwise convolution and pointwise convolution, which are mainly used for feature extraction. A convolution kernel of depthwise convolution is responsible for a channel, and a channel is convolved by only a convolution kernel, and this process produces exactly the same number of feature map channels as the input number. Point convolution applies standard convolution operations to the intermediate features using multiple 1 × 1 convolution cores to obtain multiple outputs of the same height and width as the input data. Finally, the output of all the convolution kernels is spliced to get its final output. Depthwise separable convolution can significantly reduce the number of parameters and computation, thus further improving the identification efficiency. Figure 3 shows the one-dimensional convolution process for DSCNN.

Suppose the size of the input data is *H* × *W* × *C*, in which H, W, and C are the height, width, and number of channels, respectively. In our experiment, W = 1. Each channel is deeply convoluted with a convolution core of 3 × 3, the parameters of which are calculated as shown in Formula (2)

$$N_{depthwise}=H\times W\times C\times 3\times 3$$ (2)

For point-by-point convolution, the output channel of the feature map generated by deep convolution is expanded by M convolution nuclei of 1 × 1 size. Formula (3) for calculating point-by-point convolution parameters is:

$$N_{po\mathrm{int}wise}=H\times W\times C\times 1\times 1\times M$$ (3)

Therefore, the calculation of depth can be divided into convolution weights of depth convolution and point convolution, shown in Formula (4)

$$N_{separable}=N_{separable}+N_{po\mathrm{int}wise}=H\times W\times C\times \left(3\times 3+M\right)$$ (4)

For standard convolution, the parameters are calculated as follows, Formula (5)

$$N_{s\tan dard}=H\times W\times C\times 3\times 3\times M$$ (5)

By comparing Formulas (4) and (5), it can be seen that the ratio of standard convolution to parameter calculations of deeply divisible convolution is (M × 9)/(M + 9) and the required parameters are reduced by the use of depthwise separable convolution compared to ordinary convolution (Hou et al., 2022).

The most important thing is that the depthwise separable convolution can change the channel and region of the previous common convolution operation. Convolution first considers the region and then the channel. The separation of channels and regions was achieved.

#### 2.3.4 Bi-directional long short term memory (Bi-LSTM)

**FIGURE 4:**
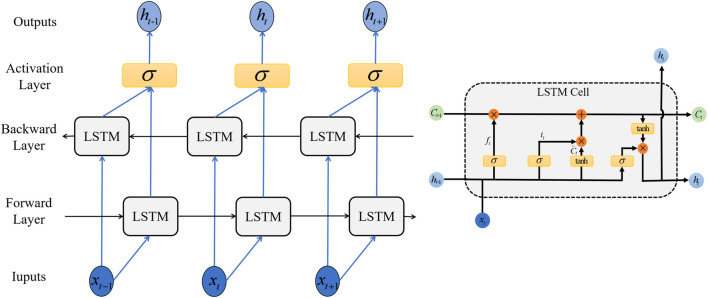
Bi-LSTM Structure and LSTM cell structure.

A recurrent Neural Network (RNN) is a neural network that can analyze data sequences relying on previous calculation result. This leads to the possibility of gradient disappearance and the explosion of RNN, limiting its availability in long input sequence analysis. Therefore, we used Bi-directional Long Short Term Memory Network (Bi-LSTM) in this paper, which is an improved circular neural network and can learn long term information. Figure 4 shows the structure of the Bi-LSTM network and LSTM cell structure.

The special structure of LSTM network avoids the long term dependencies caused by complex repetitive chain modules. The parameters of LSTM are shown respectively in Formulas (6–11). The LSTM network consists of input gates, forgotten gates, and output gates that update and delete information into storage units. Cell state *C*_t_ and “gate” structures are key parameters of LSTM. In particular, the forgotten Gate decides whether or not to delete past information from the cell state. In these formulas, *x*_*t*_ and *h*_t-1_ are determined by the output of hidden layer and sigmoid activation function *σ*, and *W* represents the corresponding weight matrix. *b* is network bias. *f*_t_, *i*_t_ and *O*_t_ are the state of the forgotten gate, the input gate and the output gate, respectively. *C*_t_ stands for the temporary state of input at the moment of *t*, *tanh* () is the unit output.

$$f_{t}=\sigma \left(w_{f}\times \left[h_{t-1},x_{t}\right]+b_{f}\right)$$ (6)

$$i_{t}=\sigma \left(w_{i}\times \left[h_{t-1},x_{t}\right]+b_{i}\right)$$ (7)

$${\tilde{C}}_{t}=\tanh\left(w_{c}\times \left[h_{t-1},x_{t}\right]+b_{c}\right)$$ (8)

$$C_{t}=f_{t}\times C_{t-1}+i_{t}\times {\tilde{C}}_{t}$$ (9)

$$o_{t}=\sigma \left(w_{o}\times \left[h_{t-1},x_{t}\right]+b_{o}\right)$$ (10)

$$h_{t}=o_{t}\times \tanh\left(C_{t}\right)$$ (11)

In a one-way long short term memory network, the network does not consider the follow-up information and drives the follow-up information by learning from the previous information. However, in many cases, prediction often requires sufficient contextual information to extract key features. Unlike LSTM, which consists of two LSTMs that send opposing messages in a looped diagram, the network connects not only the past but also the future, making the model more robust. Bi-LSTM processes two LSTMs, which consider the values before and after input and then combine the outputs. In this way, for each piece of data, LSTM can learn about the impact of previous data and the impact of subsequent data. Therefore, unlike normal LSTM, the Bi-LSTM calculation is performed by the values of two layers, which can learn long-term dependencies and effectively compensate for disappearing gradients (Zheng and Chen, 2021).

#### 2.3.5 Attention mechanism

Attention mechanism is a resource allocation mechanism that mimics the attention of the human brain, which focuses on areas that need concentration at a particular moment, reducing or even ignoring attention to other areas for more detailed information. For a given target, by generating a weighted summation of the input to identify which features in the input are important for the target and which are not. The attention mechanism improves the accuracy of the model by paying sufficient attention to the key information and highlighting the impact of the key information by means of probability distribution. It can effectively improve the time series too long to lose information and replace the original method of randomly assigning weights with probabilities.

### 2.4 Evaluation indexes

In this study, frequently used indicators in the classification were used to assess the validity and robustness of our framework from different perspectives including five indicators: accuracy, precision, recall, F1-score, and Matthews’ correlation coefficient. These evaluation indicators were defined as follows: TP, FN, TN, and FP represent true positive, false negative, true negative, and false positive, respectively.

$$accuracy=\frac{right}{all}$$ (12)

$$precision=\frac{TP}{TP+FP}$$ (13)

$$recall=\frac{TP}{TP+FN}$$ (14)

$$F1-score=\frac{2}{\frac{1}{precision}+\frac{1}{recall}}$$ (15)

$$MCC=\frac{TP\times TN-FP\times FN}{\sqrt{\left(TP+FP\right)\left(TP+FN\right)\left(TN+FP\right)\left(TN+FN\right)}}$$ (16)

## 3 Experimental results and analysis

### 3.1 Experimental setup

In this experiment, to prevent a high correlation of EEG data and to make the results more reasonable and accurate, the experimental on SEED dataset was divided into 80% (12 individuals, training set) and 20% (3 individuals, testing set), and the experimental on DREAMER dataset was divided into18 individuals training set and 5 individuals testing set. The experiment conducted 150 rounds of training using the Adam optimizer, with the Batch size set at 1,024. To further verify the performance of the model, a 10-fold cross-validation experiment was performed which used mean values as the model evaluation criteria. While ensuring the same data distribution between the training and test sets, setting all pre-training data to the same random seed, randomly scrambling, and transferring to the network model. CDBA and other comparative network models were implemented and modeled using the same parameter settings on GeForce RTX 2080Ti.

### 3.2 Single test result of CDBA model

**FIGURE 5:**
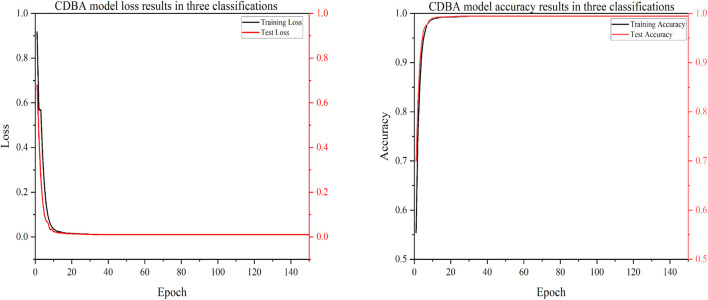
Test results of CDBA model based on three-classifications.

**FIGURE 6:**
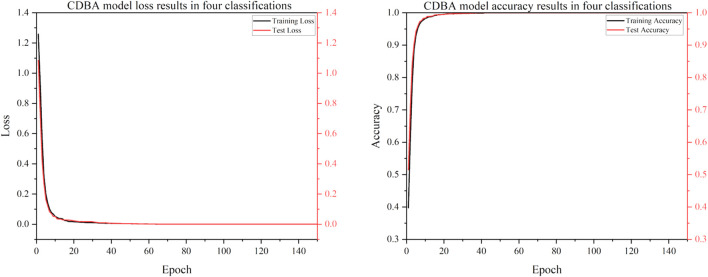
Test results of CDBA model based on four-classifications.

**FIGURE 7:**
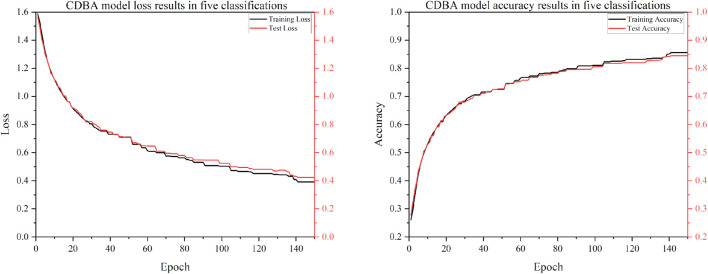
Test results of CDBA model based on five-classifications.

To verify the classification performance of the proposed CDBA model in EEG emotion detection, the model was combined with Deep Neural Network (DNN), Convolution Neural Network (CNN), Gated Recurrent Unit (GRU), Recurrent Neural Network (RNN), Long Short Term Memory Network (LSTM) and Bi-directional Long Short Term Memory Network (Bi-LSTM). And their combined models CNN-RNN, CNN-LSTM, CNN-Bi-LSTM, DSCNN-RNN, DSCNN-LSTM and DSCNN-Bi-LSTM. 1D Convolutional Auto-Encode (CAE) is composed of two convolutional layers replacing the fully connected layers, and the symbols of the input are down-sampled to provide a potential representation of smaller dimensions. 1D InceptionV1 is compared. 1D InceptionV1 is the replacement of InceptionV1 two-dimensional convolution nuclei with one-dimensional convolution nuclei. In addition to comparing the model to other deep learning models, we compared it to four popular traditional machine learning types: Adaboost, Bayes, Decision Tree and XGBoost. Traditional machine learning methods have also been widely used in many computer fields. Traditional machine learning feature extraction relies on manual methods that are simple, efficient and explainable for particularly simple tasks. The advantage of deep learning is that features can be extracted automatically. The CDBA model validates test set results on a three, four and five category task, as shown in Figures 5–7. As can be seen from these figures, the CDBA model has fast convergence speed and good performance.

The results of the validation experiments for the triple-category and four-category test sets are shown in Tables 3, 4, with three classification labels representing positive, neutral and negative emotions, respectively. Four categories of labels represent feelings of happiness, sadness, fear and neutrality. Of all the comparison models, the CDBA presented here performed best in three categories, with 99.44% accuracy, 99.45% precision, 99.44% recall, 99.44% F1-score, and 99.17% MCC. The four categories were 99.99% accuracy, 99.98% precision, 99.98% recall, 99.98% F1-score and 99.98% MCC. The 1D CAE model was 95.92% accuracy, 95.92% precision, 95.92% recall, 95.91% F1-score, and 93.88% MCC for all three categories of tasks. The DSCNN-LSTM model had 88.87% accuracy, 88.87% precision, 88.87% recall, 88.87% F1-score and 85.17% MCC across the four categories. These two models are second only to CDBA models in the three and four classification tasks. Of the four traditional machine learning models, XGBoost and Decision Tree performed best in the three and four categories, respectively, while Bayes performed worst in the three and four categories.

**TABLE 3:** The performance of CDBA model on three-category of task test sets.

Methods	Accuracy (%)	Precision (%)	Recall (%)	F1-score (%)	MCC(%)
DNN	68.15	68.34	68.15	68.13	52.32
CNN	80.61	80.71	80.61	80.59	70.97
GRU	68.95	68.97	68.95	68.83	53.53
RNN	67.53	67.40	67.53	67.14	51.54
LSTM	88.77	88.77	88.77	88.77	83.15
Bi-LSTM	92.08	92.12	92.08	92.08	88.16
CNN-RNN	77.62	77.66	77.62	77.56	66.49
CNN-LSTM	94.69	94.70	94.69	94.69	92.04
CNN-Bi-LSTM	93.10	93.16	93.10	93.09	89.69
DSCNN-RNN	72.43	73.09	72.43	72.23	59.09
DSCNN-LSTM	94.03	94.04	94.03	94.03	91.05
DSCNN-Bi-LSTM	91.10	91.30	91.10	91.08	86.76
1D CAE	95.92	95.92	95.92	95.91	93.88
1D InceptionV1	87.72	87.89	87.72	87.70	81.68
Adaboost	54.29	55.03	54.29	53.99	31.86
Bayes	40.95	42.97	40.95	35.88	13.77
Decision Tree	79.38	81.06	79.38	79.47	69.78
XGBoost	95.12	95.21	95.12	95.12	92.73
**CDBA (proposed model)**	**99.44**	**99.45**	**99.44**	**99.44**	**99.17**

**TABLE 4:** The performance of CDBA model on four-category of task test sets.

Methods	Accuracy (%)	Precision (%)	Recall (%)	F1-score (%)	MCC(%)
DNN	52.12	53.56	52.12	52.06	36.56
CNN	64.21	64.59	64.21	64.20	52.38
GRU	49.44	49.42	49.44	49.29	32.65
RNN	49.12	49.15	49.12	48.66	32.37
LSTM	75.83	75.93	75.83	75.78	67.83
Bi-LSTM	84.13	84.27	84.13	84.13	78.89
CNN-RNN	56.87	57.72	56.87	56.91	42.72
CNN-LSTM	87.68	87.71	87.68	87.68	83.59
CNN-Bi-LSTM	85.43	85.52	85.43	85.45	80.59
DSCNN-RNN	55.35	55.48	55.35	54.75	40.78
DSCNN-LSTM	88.87	88.87	88.87	88.87	85.17
DSCNN-Bi-LSTM	84.08	84.08	84.08	84.08	78.78
1D CAE	87.29	87.29	87.29	87.29	83.06
1D InceptionV1	78.06	78.17	78.06	78.07	70.77
Adaboost	37.49	37.52	37.49	37.41	16.69
Bayes	26.10	30.44	26.10	17.39	24.6
Decision Tree	88.46	88.63	88.46	88.50	84.65
XGBoost	87.23	87.34	87.23	87.24	82.99
**CDBA (proposed model)**	**99.99**	**99.98**	**99.98**	**99.98**	**99.98**

The experimental results of five classifications on DREAMER dataset are shown in Table 5, and the CDBA model is still the best performance, with 84.49% accuracy, 84.81% precision, 84.07% recall, 84.38% F1-score, and 80.38% MCC for all five categories of tasks. Bayes method performs the worst. The results show that the CDBA model proposed in this paper is superior to other models. The three-block parallel structure can extract the characteristics of the input signal simultaneously, and can extract the temporal and spatial features from the original features, thus to improve the accuracy of the model.

**TABLE 5:** The performance of CDBA model on five-category of task test sets.

Methods	Accuracy (%)	Precision (%)	Recall (%)	F1-score (%)	MCC(%)
DNN	30.14	32.38	28.32	27.75	10.96
CNN	34.16	37.38	31.95	31.38	16.08
GRU	42.18	47.12	40.31	40.87	26.72
RNN	38.28	42.05	35.89	36.06	21.43
LSTM	37.71	42.92	35.85	35.62	21.39
Bi-LSTM	42.21	44.57	40.16	40.40	26.59
CNN-RNN	50.81	53.77	49.04	49.65	37.88
CNN-LSTM	62.10	63.31	61.26	61.92	52.09
CNN-Bi-LSTM	67.55	69.59	66.46	67.44	59.01
DSCNN-RNN	38.67	41.61	36.48	36.41	22.13
DSCNN-LSTM	38.27	40.17	37.23	36.93	22.34
DSCNN-Bi-LSTM	38.27	40.17	37.23	36.93	22.34
1D CAE	65.68	66.31	65.66	65.82	56.83
1D InceptionV1	32.86	38.14	30.77	30.24	14.47
Adaboost	34.66	34.63	32.70	32.64	16.76
Bayes	22.53	23.94	22.28	15.96	3.96
Decision Tree	65.66	67.11	65.00	65.72	56.63
XGBoost	74.97	76.39	74.40	75.14	68.41
CDBA	84.49	84.81	84.07	84.38	80.38

### 3.3 Ten-fold cross validation result of CDBA model

**FIGURE 8:**
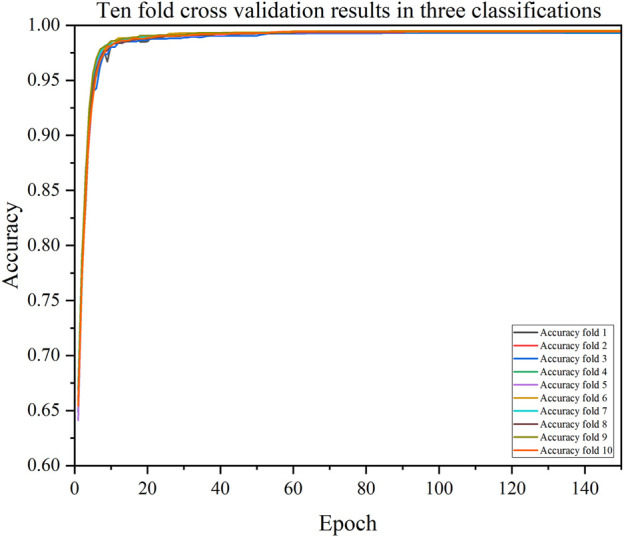
The accuracy of CDBA model based on ten-fold cross-validation three-classification task.

**FIGURE 9:**
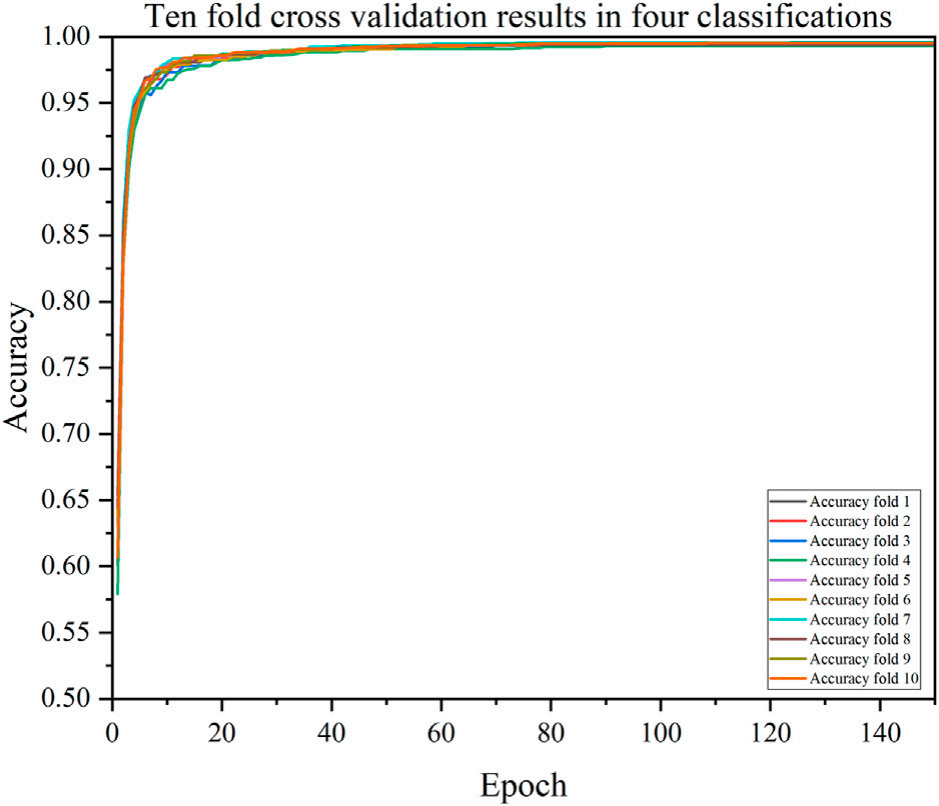
The accuracy of CDBA model based on ten-fold cross-validation four-classification task.

**FIGURE 10:**
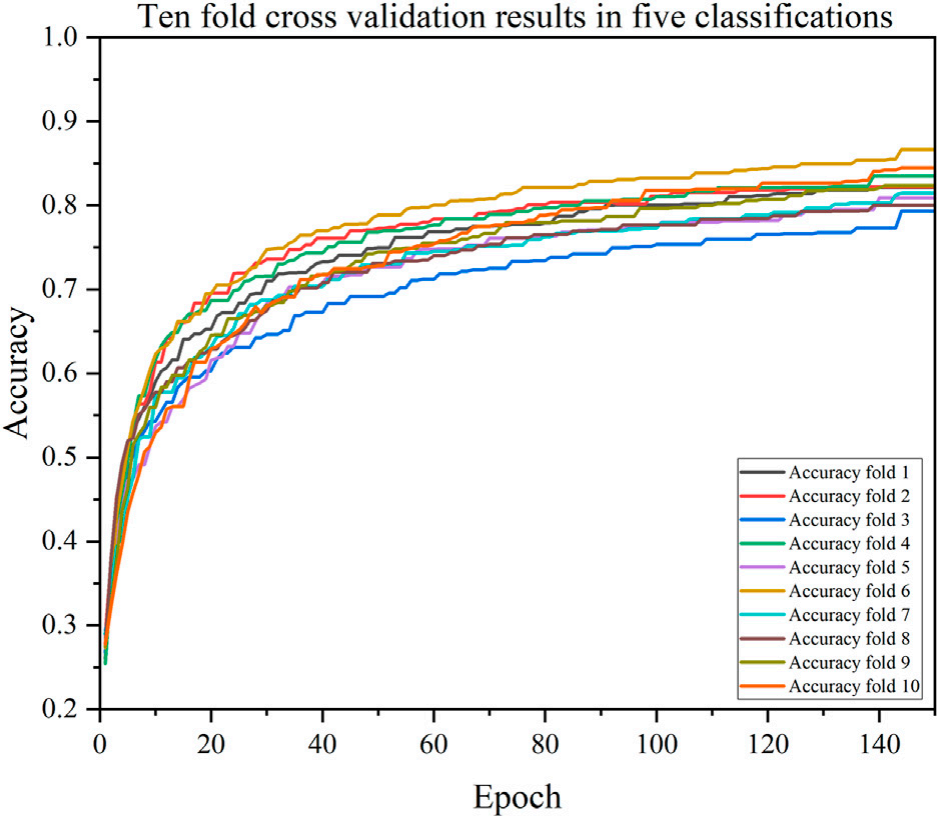
The accuracy of CDBA model based on ten-fold cross-validation five-classification task.

We also validated the performance of each model with a 10-fold cross-validation. The 10 cross-validation sessions divided the dataset into 10 segments, with 9 as the training set, 1 as the test set, and the mean value of the 10 cross-validation sessions as an estimate of the algorithm’s accuracy. The results of the CDBA model with 10-fold cross-validation are respectively shown in Figures 8–10. Tables 6–8 show the results of the average 10-fold cross-validation. Of all the comparison models, the CDBA model presented here continues to perform best, with 99.40% accuracy, 99.41% precision, 99.40% recall, 99.40% F1-score, and 99.11% MCC across all three categories of tasks. The four categories of tasks were 99.51% accuracy, 99.51% precision, 99.51% recall, 99.51% F1-score, and 99.36% MCC. The five categories of tasks were 82.30% accuracy, 82.50% precision, 81.95% recall, 82.16% F1-score, and 77.63% MCC. The CNN-LSTM model was 93.28% accuracy, 93.29% precision, 93.27% recall, 93.28% F1-score, and 89.92% MCC for all three categories of tasks. The 1D CAE model was 83.20% accuracy, 83.26% precision, 83.20% recall, 83.20% F1-score, and 77.63% MCC for all four categories of tasks. The Decision Tree model was 77.81% accuracy, 77.60% precision, 77.55% recall, 77.58% F1-score, and 71.96% MCC for all five categories of tasks. These two models were second only to CDBA models in the three, four and five classification tasks. The worst-performing model was the Bayes model, with 41.79% accuracy, 42.23% precision, 41.79% recall, F1-score of 38.82%, and MCC of 13.75% in the three-category task. The four-category task had an accuracy rate of 25.77%, precision of 28.84%, recall rate of 25.77%, F1-score of 17.34%, and MCC score of 16.60%. The five-category task had an accuracy rate of 21.88%, precision of 21.19%, recall rate of 21.11%, F1-score of 13.76%, and MCC score of 2.24%. As can be seen from the results of single validation and 10-fold cross-validation, the model presented in this paper has the best performance of all comparison models.

**TABLE 6:** The performance of CDBA model based on ten-fold cross-validation (three-classification task).

Methods	Accuracy (%)	Precision (%)	Recall (%)	F1-score (%)	MCC(%)
DNN	66.42	66.87	66.42	66.33	49.86
CNN	73.86	74.07	73.86	73.82	60.92
GRU	66.35	66.72	66.35	66.17	49.80
RNN	66.05	66.31	66	65.93	49.29
LSTM	87.69	87.76	87.69	87.68	81.58
Bi-LSTM	92.68	92.70	92.68	92.67	89.03
CNN-RNN	73.03	73.31	73.02	72.98	59.70
CNN-LSTM	93.28	93.29	93.27	93.28	89.92
CNN-Bi-LSTM	92.17	92.19	92.17	92.17	88.28
DSCNN-RNN	70.57	70.93	70.58	70.49	56.07
DSCNN-LSTM	92.96	92.98	92.96	92.96	89.46
DSCNN-Bi-LSTM	89.74	89.77	89.75	89.74	84.64
1D CAE	92.07	9,212	92.06	82.88	88.13
1D InceptionV1	82.27	82.60	82.27	82.25	73.58
Adaboost	52.63	53.38	52.64	52.35	29.35
Bayes	41.79	42.23	41.79	38.82	13.75
Decision Tree	81.08	81.08	81.08	81.08	71.62
XGBoost	90.69	90.91	90.69	90.69	86.14
**CDBA (proposed model)**	**99.40**	**99.41**	**99.40**	**99.40**	**99.11**

**TABLE 7:** The performance of CDBA model based on ten-fold cross-validation (four-classification task).

Methods	Accuracy (%)	Precision (%)	Recall (%)	F1-score (%)	MCC(%)
DNN	49.85	50.37	49.85	49.70	33.32
CNN	62.46	62.77	62.46	62.43	50.05
GRU	47.80	48.35	47.81	47.67	30.59
RNN	46.35	47.08	46.33	45.57	28.89
LSTM	74.21	74.33	14.11	74.21	65.66
Bi-LSTM	81.49	81.59	81.50	81.49	75.36
CNN-RNN	54.99	55.74	54.99	54.73	40.29
CNN-LSTM	83.65	83.70	83.64	83.65	78.21
CNN-Bi-LSTM	82.20	82.25	82.20	82.20	76.29
DSCNN-RNN	54.99	55.74	54.99	54.73	40.29
DSCNN-LSTM	81.15	81.18	81.15	81.14	74.88
DSCNN-Bi-LSTM	79.98	80.08	79.97	79.97	73.34
1D CAE	83.20	83.26	83.20	83.20	77.63
1D InceptionV1	73.31	73.96	73.30	73.24	64.64
Adaboost	35.93	36.00	35.93	35.82	14.61
Bayes	25.77	28.84	25.77	17.34	16.60
Decision Tree	74.10	74.11	74.10	74.10	65.47
XGBoost	80.57	80.81	80.57	80.61	74.15
**CDBA (proposed model)**	**99.51**	**99.51**	**99.51**	**99.51**	**99.36**

**TABLE 8:** The performance of CDBA model based on ten-fold cross-validation (five-classification task).

Methods	Accuracy (%)	Precision (%)	Recall (%)	F1-score (%)	MCC(%)
DNN	30.64	66.04	26.84	24.79	10.24
CNN	34.23	35.59	30.94	30.14	15.56
GRU	40.54	42.47	38.31	38.51	24.18
RNN	37.23	39.82	34.57	31.40	19.77
LSTM	36.87	39.24	34.62	34.45	19.51
Bi-LSTM	42.81	45.55	40.72	41.02	27.32
CNN-RNN	46.68	48.04	45.06	45.58	32.24
CNN-LSTM	59.14	59.74	58.51	58.79	49.41
CNN-Bi-LSTM	66.93	67.69	66.39	66.70	58.27
DSCNN-RNN	39.57	41.38	37.30	36.37	22.55
DSCNN-LSTM	58.38	59.23	57.50	57.95	47.31
DSCNN-Bi-LSTM	58.30	59.45	57.43	57.72	47.19
1D CAE	66.55	67.36	65.90	66.28	57.76
1D InceptionV1	33.34	38.12	30.07	28.72	14.54
Adaboost	32.95	32.72	29.73	28.87	13.72
Bayes	21.88	21.19	21.11	13.76	2.24
Decision Tree	77.81	77.60	77.55	77.58	71.96
XGBoost	69.93	71.64	68.93	69.88	61.90
**CDBA (proposed model)**	**82.30**	**82.50**	**81.95**	**82.16**	**77.63**

### 3.4 Ablation experiment

To validate the proposed CDBA model, we performed ablation experiments on SEED and DREAMER datasets. Feature extraction is a multi-channel structure, which is the most important module in the whole model, so only the feature extraction module is changed and the rest of the module remains the same. Results from the SEED dataset ablation experiments are shown in Table 9. It can be seen that the classification performance of the model on both datasets shows the same trend in channel selection. The Block3 model performs best in single-channel triage and quadrangle tasks based on ten-fold cross-validation, due to BI-LSTM’s expertise in extracting time-series signature data such as EEG. Block1 and Block2 perform best when combined with Block3 in dual channels, as it extracts spatiotemporal features simultaneously. Combining these two features, emotional recognition models perform better. Ablation experimental performance in the DREAMER dataset was similar to that in the SEED dataset as shown in Table 10, but the Block1 + Block2 model performed best in the two-channel model, suggesting that spatial features were more pronounced for the DREAMER dataset. However, our model is still the best of all ablation experimental comparison models, which further proves the validity of the proposed method.

**TABLE 9:** Ablation experiments of CDBA model based on ten-fold cross-validation (three-classification task) and (four-classification task).

Data set	Methods	Accuracy (%)	Precision (%)	Recall (%)	F1-score (%)	MCC(%)
SEED	Block1	99.16	99.17	99.16	99.16	98.75
SEED	Block2	98.72	98.73	98.72	98.72	98.09
SEED	Block3	99.32	99.33	99.32	99.32	98.99
SEED	Block1 + Block2	98.58	98.58	98.57	98.58	97.87
SEED	Block1 + Block3	99.38	99.39	99.38	99.38	99.08
SEED	Block2 + Block3	99.35	99.35	99.35	99.35	99.03
SEED	**CDBA**	**99.40**	**99.41**	**99.40**	**99.40**	**99.11**
SEED-IV	Block1	98.92	98.93	98.92	98.92	98.57
SEED-IV	Block2	97.85	97.86	97.85	97.85	97.14
SEED-IV	Block3	99.24	99.24	99.24	99.24	99.00
SEED-IV	Block1 + Block2	97.72	97.73	97.72	97.72	96.97
SEED-IV	Block1 + Block3	99.34	99.34	99.34	99.34	99.12
SEED-IV	Block2 + Block3	99.26	99.26	99.26	99.26	99.02
SEED-IV	**CDBA (proposed model)**	**99.51**	**99.51**	**99.51**	**99.51**	**99.36**

**TABLE 10:** Ablation experiments of CDBA model based on ten-fold cross-validation (five-classification task).

Data set	Methods	Accuracy (%)	Precision (%)	Recall (%)	F1-score (%)	MCC(%)
DREAMER	Block1	68.14	68.42	67.79	67.97	59.75
DREAMER	Block2	61.98	63.45	61.30	61.84	52.00
DREAMER	Block3	69.67	69.93	69.21	69.50	61.68
DREAMER	Block1 + Block2	69.98	70.36	69.70	69.83	62.12
DREAMER	Block1 + Block3	68.83	69.22	68.56	68.71	60.68
DREAMER	Block2 + Block3	69.06	69.65	68.53	68.88	60.89
DREAMER	**CDBA (proposed model)**	**82.30**	**82.50**	**81.95**	**82.16**	**77.63**

## 4 Conclusion

Automatic emotion recognition is an important application area of artificial intelligence. In this study, a multi-branching feature fusion model based on an attention mechanism is proposed for EEG emotional recognition network. The model framework includes spatial feature extraction based on CNN and DSCNN, temporal feature extraction based on Bi-LSTM, and feature weight allocation based on attention mechanisms, and is then classified in a fully connected layer. The method requires only normalized preprocessing of the raw data, which is then fed into the CDBA model to obtain the predicted results. For the three-class task, the accuracy of the single test set was 99.44%, for the four-class task, the accuracy of the single test set was 99.99%, and for the five-class task, the accuracy of the single test set was 84.49%. The average ten-fold cross-validation accuracy of the method was 99.40% for three classifications, 99.51% for four classification tasks and 82.30% for five classification tasks. The experimental results show that the proposed multi-channel feature fusion method has better accuracy than other single-channel model and traditional machine learning model. This is because the proposed CDBA model for EEG emotion classification can simultaneously extract low-level spatial features, high-level spatial features, and time-series features, and filter out the features with the most significant expression of emotion through attention mechanisms. And from the experimental results, it can be seen that the model combining spatial features and time features will get better results than the single model. Therefore, the CDBA model proposed in this paper is the most suitable for emotion prediction compared with other models. Physiological signals like EEG have the advantages of universality, spontaneity, and difficulty in camouflage (Zali-Vargahan et al., 2023), and human cognitive behavior and mental activity have a strong correlation with EEG signals. Therefore, physiological signals are a good choice to recognize emotions (Liu et al., 2020). The CDBA model proposed in this paper provides a new idea for decoding human emotions based on EEG, and can also be used for other EEG classification tasks, such as sleep phase classification and motor imagination. The model needs only simple preprocessing to obtain high accuracy, and can be easily transplanted into EEG equipment. In the future, we plan to identify and classify human emotions more accurately by combining EEG, ECG, and EMT signals.

## Data Availability

The original contributions presented in the study are included in the article/Supplementary Material, further inquiries can be directed to the corresponding authors.
